# Selective chemoattraction of the benthic diatom *Seminavis robusta* to phosphate but not to inorganic nitrogen sources contributes to biofilm structuring

**DOI:** 10.1002/mbo3.694

**Published:** 2018-07-22

**Authors:** Karen Grace V. Bondoc, Christine Lembke, Wim Vyverman, Georg Pohnert

**Affiliations:** ^1^ Institute for Inorganic and Analytical Chemistry Bioorganic Analytics Friedrich‐Schiller‐Universität Jena Jena Germany; ^2^ Max Planck Institute for Chemical Ecology Jena Germany; ^3^ Laboratory of Protistology and Aquatic Ecology Department of Biology University Gent Gent Belgium

**Keywords:** algae, biofilm biology, growth and survival, microbial behavior, nutrient acquisition

## Abstract

Diatoms frequently dominate marine and freshwater biofilms as major primary producers. Nutrient resources in these biofilms are patchily distributed and fluctuate dynamically over time. We recently reported that this spatially and temporally structured environment can be exploited by motile diatoms that use chemoattraction to dissolved silicate (dSi) under Si starvation. Here, we show that the behavioral response of diatoms is more complex and selective as cells are also responding to gradients of dissolved phosphate (dP) when starved in this nutrient. In contrast, neither nitrate nor ammonium (dN) triggers an attractive response under nitrogen limitation. Video monitoring and movement pattern analysis of the model diatom *Seminavis robusta* revealed that dP attraction is mediated by a combined chemokinetic and chemotactic response. After locating nutrient hotspots, the microalgae slow down and recover from the limitation. The fastest recovery in terms of growth was observed after dSi limitation. In agreement with the lack of directional response, recovery from dN limitation was slowest, indicating that no short‐term benefit would be drawn by the algae from the location of transient hotspots of this resource. Our results highlight the ability of diatoms to adapt to nutrient limitation by active foraging and might explain their success in patchy benthic environments.

## 1 INTRODUCTION

Inorganic nutrients are dynamically and patchily distributed in the ocean and control primary production in this environment (Falkowski, Barber, & Smetacek, 1998). Particularly in biofilms, nutrients like silicate and phosphate are found in hotspots due to the dissolution of mineral particles (Paytan & McLaughlin, 2007). Small‐scale horizontal and vertical gradients of nutrients range from micrometers to several meters (Bernhard, Visscher, & Bowser, 2003; Stockdale, Davison, & Zhang, 2009; Underwood & Kromkamp, 1999). As a consequence, the benthic environment, one of the most productive marine ecosystems (Underwood & Kromkamp, 1999), is characterized by heterogeneity in biomass and species distribution. Pennate diatoms are pivotal for benthic biofilm formation in marine and freshwater habitats where they dominate as primary producers and contribute to sediment stabilization (Underwood & Kromkamp, 1999). In this environment, they permanently interact with other microorganisms including marine bacteria (Bruckner, Rehm, Grossart, & Kroth, 2011), and have to cope with fluctuations in light and nutrient availability (Magni & Montani, 2006; Moerdijk‐Poortvliet et al., 2018; Stocker, 2012). Therefore, microalgae have developed adaptation mechanisms and can, for example, take up and store dissolved phosphorous or nitrogen under replete conditions (Brembu, Muhlroth, Alipanah, & Bones, 2017; Lin, Litaker, & Sunda, 2016; Rogato et al., 2015). Some benthic diatoms including the model species *Seminavis robusta* are motile and move in a gliding manner by excreting extracellular polymeric substances (EPS) from elongated slits (raphae) in their biomineralized cell wall (Molino & Wetherbee, 2008). Pseudopod‐like structures are formed, which alternately press on the substrate, and allow changes in direction resulting in a backward and forward movement with sporadic turns (Wang, Cao, Du, & Chen, 2013). This motility enables vertical relocation guided by phototaxis to optimize light conditions (McLachlan, Brownlee, Taylor, Geider, & Underwood, 2009). Given the highly dynamic light availability governed by photoperiods and tidal cycles, directed motility can substantially increase the fitness of benthic diatoms (Consalvey, Paterson, & Underwood, 2004; Perkins et al., 2010). Moreover, this motility can also assist in the location of local nutrient hotspots as we recently reported in the case of the active foraging behavior of silicate‐limited *S. robusta* that locates dissolved silicate (dSi) hotspots through a simultaneous tactic and kinetic mechanism (Bondoc, Heuschele, Gillard, Vyverman, & Pohnert, 2016). In this diatom, motility and chemoreception are also essential for the finding of mating partners for sexual reproduction within pheromone gradients (Gillard et al., 2013).

While dSi utilization as a macronutrient is rather unique in diatoms, as they use it mainly for their biomineralized cell wall (Kröger, Lorenz, Brunner, & Sumper, 2002), nitrogen and phosphorus are essential resources for biomacromolecule production in all phyla. Hence, ammonium, nitrate, nitrite, and phosphate are limiting diatom growth as well (Lin et al., 2016; Moore et al., 2008; Paytan & McLaughlin, 2007). By combining physiological and behavioral studies, we show that active foraging of benthic diatoms under phosphorus starvation can compensate for local minima of this resource. In comparative attraction assays we observed active searching behavior for dP, but not for dN hotspot sources. The response upon attraction toward dP was comparable to the ones found for dSi and pheromone attraction, albeit accumulation of the cells around point sources of the nutrients occurred slower (Bondoc, Heuschele, et al., 2016; Bondoc, Lembke, Vyverman, & Pohnert, 2016). We suggest that motile benthic diatoms can enhance their nutrient encounter by actively searching for nutrient hotspots and, thereby, outcompete immotile species. Thus, our study underlines the high success of these organisms in their benthic environment.

## 2 EXPERIMENTAL PROCEDURES

### 2.1 Culturing conditions and microscopy

We used the *S. robusta* strain PONTON36 (24.0 ± 0.6 μm), which is accessible at the Belgian Coordinated Culture Collection of Microorganisms (bccm.belspo.be, accession number DCG 0462). Cultivation was performed in 40‐ml TC flasks (Sarstedt, Germany) in buffered artificial sea water (ASW) modified after Maier & Calenberg (1994) at 18°C in a 12:12 hr light:dark regime with cool‐white fluorescent lamps at approximately 35 μmol photons m^−2^ s^−1^. The nutrient concentrations in the medium were set at 621 μM NaNO_3_ (VWR Chemicals, Leuven, Belgium), 15 μM K_2_HPO_4_ (Roth, Karlsruhe, Germany), and 246 μM Na_2_SiO_3_·9 H_2_O (Sigma‐Aldrich, Steinheim, Germany).

### 2.2 Monitoring diatom growth and nutrient analysis

Stock cultures were grown for 7 days to reach early stationary phase before one tenth of the initial culture was diluted into 25‐ml fresh ASW medium. The growth of experimental cultures (*n* = 12) was monitored under an inverted microscope by taking triplicate photos of three randomly chosen culture flasks every day. The cell density was calculated by counting the number of cells per picture semiautomatically in Fiji (http://fiji.sc/Fiji) (Schindelin et al., 2012). Every second day, the medium of three replicates was harvested for nutrient analysis by sterile filtration (Filtropur S 0.2, Sarstedt, Nümbrecht) and stored at −20°C until measurement. Silicate and phosphate concentrations were determined by standard photospectrometric methods (Strickland & Parsons, 1972). The concentrations of nitrate, nitrite, and ammonia were measured by flow analysis and photospectrometric detection according to ISO 11732‐2005 and ISO 13395‐1996 by the “Routine Measurements and Analysis of Environmental Samples” laboratory team at the Max Planck Institute for Biogeochemistry Jena, Germany.

### 2.3 Diatom growth under nutrient starvation and recovery experiment

**Figure 1:**
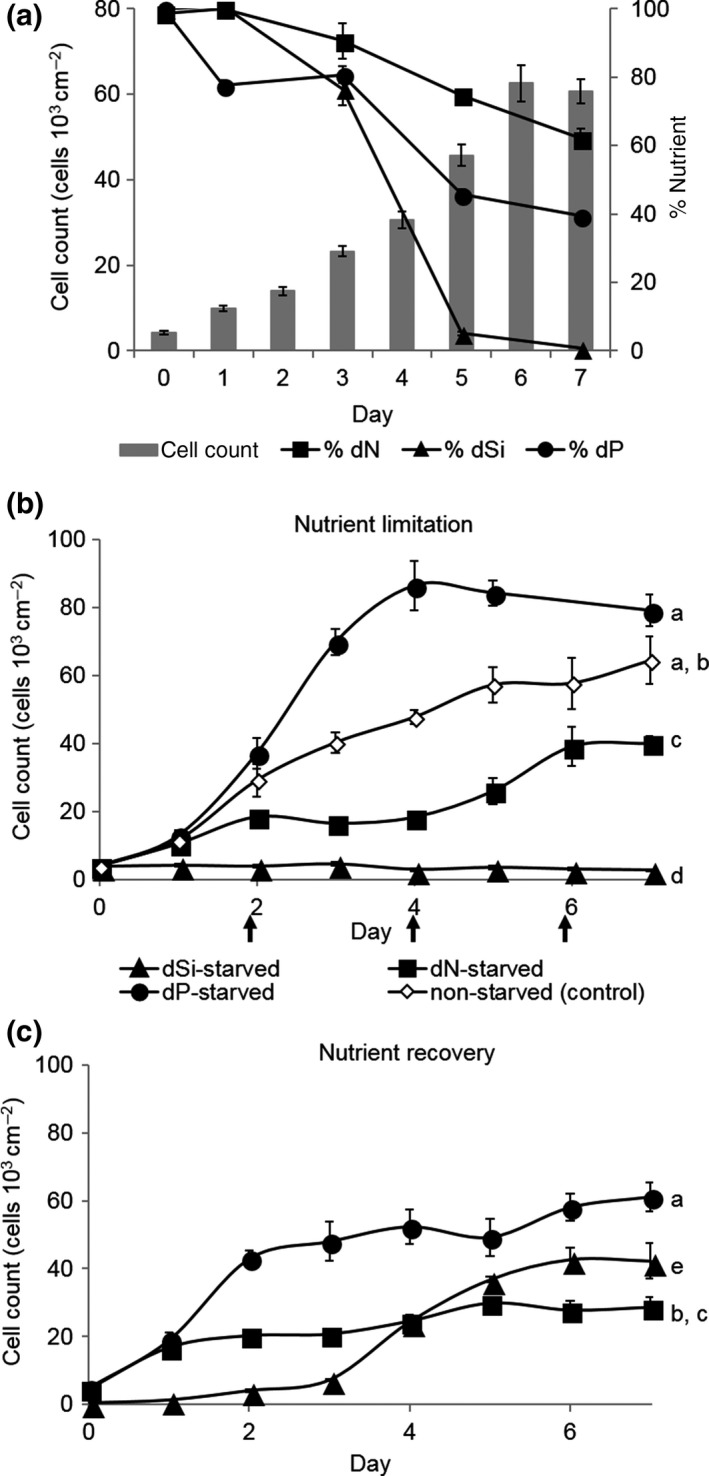
Growth and nutrient dynamics in *Seminavis robusta* batch cultures. All data points are presented as mean ± *SEM* (*n* = 3). (a) As cells reach late exponential phase, dSi was depleted in the medium (100% = 246 μM). Meanwhile, concentrations of both dN (100% = 621 μM) and dP (100% = 15 μM) remained at ~60% and ~40%, respectively (*n* = 3). (b) Cells in stationary phase were transferred to medium depleted with dN, dSi, dP, or medium with all nutrients (nonstarved as control) and growth was observed for 1 week. The medium was exchanged every 2 days (indicated by arrows) for dP‐ and dN‐starved cells. dSi‐starved cells showed no growth as compared with dN‐ and dP‐starved cells and control (linear mixed effects (LME) modeling with pairwise Tukey's HSD for all *p* < 0.001, *n* = 3). Cells limited in dN continued to grow, presumably due to stored nutrients while dP‐limited cells reached maximum cell counts after day 4. Treatments with same letters are not statistically significant from each other. Statistical tests of this set were performed together with data from Figure 1c. (c) After starvation (3 days for dSi, 5 days for dP, and 12 days for dN), aliquots of starved cells were transferred to nutrient‐replete medium. dSi‐ and dP‐starved cells showed a fast recovery compared to dN‐starved cells (LME with pairwise Tukey's HSD, for dSi vs. dN:* p *< 0.001, for dP vs. dN:* p* = 0.001). Treatments with same letters are not statistically significant from each other. Statistical tests of this set were performed together with data from Figure 1b

For monitoring cell growth under nutrient limitation, experimental cultures were inoculated into 25‐ml ASW where no dSi, dN, or dP was added, but which was supplemented with all other nutrients. As a control, full ASW medium was used. Cell growth was monitored as described above (*n* = 3) and the dN‐ or dP‐depleted medium was exchanged every 2 days to avoid that other nutrients become limited. Due to slow growth, this exchange was not required for the dSi‐limited cultures. After 3, 5, or 12 days, 10% of the dSi‐, dP‐, or dN‐depleted cultures, respectively, were inoculated in fresh ASW and cell growth was monitored as described above (*n* = 3). A linear mixed effects (LME) model coupled with the post hoc Tukey's HSD was used to analyze the data (Figure 1, Table S2).

### 2.4 dP, dN, and dSi starvation for motility and attraction analyses

Experimental cultures were inoculated in the nutrient‐deplete medium as described above. After culturing under limited condition (3 days for dSi limitation, 5 days for dP limitation, or 12 days for dN limitation), cells were used for motility and attraction studies. Cells grown in ASW served as a control. Twenty‐four hours before video monitoring cells were scraped off from the culture flask and aliquots of 2 ml were transferred into 12‐well plates (Sarstedt, Nümbrecht, Germany).

### 2.5 Bead preparation and monitoring of chemoattraction

Bead preparation and attraction assays were modified from Bondoc, Heuschele, et al. (2016). To 100‐mg aluminum oxide beads (Merck, Darmstadt, Germany; 90 active neutral; 0.063–0.200 mm) 800 μl of 440 mM Na_2_SiO_3_·9 H_2_O, 200 μl of 670 mM NaNO_3_ or NH_4_Cl, or 800 μl of 225 mM KH_2_PO_4_ was added and dried overnight at 60°C, resulting in 1.40 nmol dSi, 0.54 nmol dN, and 0.72 nmol dP per bead, respectively. These concentrations allowed concentrations of ~44 μM from the surface of the bead to ~4 μM dP at the edge of the observation area and 100–9 μM dSi to build up (Figure S1, Bondoc, Heuschele, et al., 2016; Bondoc, Lembke, et al. 2016). Control beads were correspondingly prepared using water. Loading success and release rates were monitored as described in the supporting information. Aliquots of beads (~30 beads per well) were carefully introduced using a spatula to cells starved with dSi, dN, or dP for 3, 5, and 12 days, respectively, and attraction was monitored for 1 hr.

### 2.6 Monitoring of motility under the influence of dP

dP‐limited cells in 12‐well plates were grown for 24 hr, and the medium was exchanged with either dP‐depleted or full ASW medium. As a control, nonstarved cells were used. Motility of the cells was monitored under illumination in the microscope before and 1 hr after medium exchange by taking 60 s movies per well.

### 2.7 Video and statistical analyses

All movies were preprocessed from 100 fps to 1 fps using VirtualDub (http://virtualdub.org/) and Fiji (http://fiji.sc/). A semiautomatic tracking with spot checking and track correction to precisely determine distinct movement patterns was performed using the open‐source tracking plug‐in, TrackMate (http://fiji.sc/TrackMate) (Tinevez et al., 2017). Automatic tracking parameters of the simple LAP tracker were first optimized on an individual movie before using it for the whole experimental dataset. The open‐source statistical program R v. 3.3.1 (R Core Team, 2016) with the packages ggplot2 (Wickham, 2016) for plotting, nlme (Pinheiro, Bates, DebRoy, & Sarkar, 2015), for linear mixed effects (LME) modeling, and mgcv (Wood, 2011) for general additive mixed modeling (GAMM) was used for all data analyses. Only dP movies and their corresponding controls (*n*_movies_ = 3, *n*_cells/movie_ = 15) were evaluated as dSi movies were previously analyzed (Bondoc, Heuschele, et al., 2016) and dN movies showed no attraction. For dP experiments with beads, the observation area was divided into three concentric circles, referred to as bins (bins A–C), having a radius of 112 μm, 224 μm, and 336 μm, respectively, with the bead in the middle.

Our main data are either count data with or without trajectory analysis. We calculated parameters such as speed and sum distance of cells from track data. Cell counts were transformed either using log‐normal for nutrient starvation and recovery studies or *Z*‐standard score for cell accumulation sets, whereas speed was log + 1 transformed. Depending on the longitudinal and nesting of the experimental design, either LME or GAMM was used to fit the data. To correct for correlated data between independent variables and residual spreads, an autoregressive order 1 (AR‐1) correlated structure and a constant variance function structure (varIdent), respectively, were added to the model when necessary. For optimum model fitting, the Akaike Information Criterion (AIC) as well as residual and model plots were checked. A Wald test to determine the significance of the fitted estimates was performed on the chosen optimum model. For pairwise comparisons of treatments, either a Tukey's Honest Significance Difference (HSD) test or Least Square (LS) means test was employed as a post hoc test. The detailed model fit for each figure (Table S1), as well as results of each model (Tables S2–S6) can be found in the Supplemental Information.

## 3 RESULTS

### 3.1 Cell growth and nutrient limitation

For the systematic comparison of the effects of nutrient limitation, the growth and nutrient usage in *S. robusta* was analyzed (Figure 1). We followed *S. robusta* batch cultures in artificial seawater (ASW) and documented their nutrient use (dSi, dP, and dN in NO_3_^−^ form) over 7 days. Nutrient analysis revealed that the limiting factor was dSi, which was below the 1‐μM detection limit during the stationary phase. In contrast, dN and dP concentrations were not limiting (in stationary phase dN remained at ~60% of the initial 621‐μM concentration and dP at ~40% of the initial 15‐μM concentration) (Figure 1a). To elucidate the effects of limitation, we performed studies using modified ASW medium that was prepared without the addition of the respective nutrient under investigation. Cells starved in dSi did not grow even in the presence of excess dN and dP while cells in dP‐depleted medium continued to divide for 4 days. Cell density in dN‐starved cultures increased at a lower rate than in dP‐starved and control treatments (Figure 1b, Table S2). To fully deplete cellular nutrient stores from the cells, medium from batch cultures was exchanged with fresh medium supplemented with all nutrients except for the limiting one every other day. The treatment was repeated until no further growth was observed after 3, 5, and 12 days of starvation for dSi, dP, and dN, respectively. Aliquots of these cultures were transferred to artificial seawater containing the full amount of nutrients (ASW), and growth was monitored to determine cell recovery. After 7 days, cell densities increased 100‐, 12‐, and 6‐fold after addition of dSi, dP, and dN, respectively, highlighting survival and recovery capabilities (Figure 1c, Table S2).

### 3.2 Attraction of starved cells within nutrient gradients

**Figure 2:**
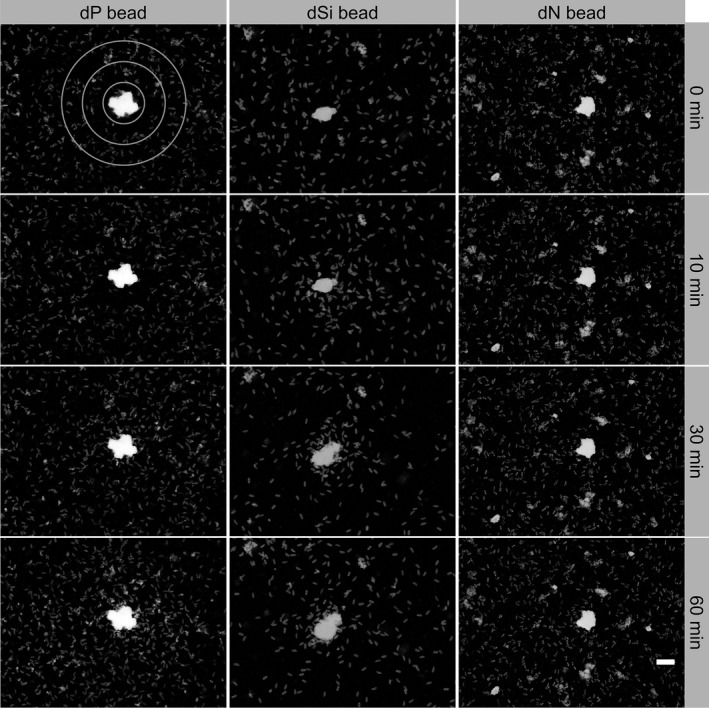
Attraction to nutrients. Response of *S. robusta* to nutrient‐loaded beads. Cells in stationary phase were transferred to either dP‐, dSi‐, or dN‐ (NH_4_^+^ or NO_3_^−^) free medium and were starved for 5, 3, or 12 days, respectively. For both dP and dN, the medium was exchanged every second day to ensure that cells are under truly limiting condition for the respective nutrient. Starved cells accumulated within ~10 min around dSi beads, ~30 min around dP beads, and no attraction was observed toward dN (here nitrate) beads. For further analysis, the observation area was divided into three bins, divided by circles with radii of 112 μm, 224 μm, and 336 μm (upper left). Scale bar corresponds to 100 μm

We tested if nutrient‐starved cells can locate hotspot sources in attraction assays. Aluminum oxide particles that do not elicit any response in the diatoms (Bondoc, Heuschele, et al., 2016) were loaded with the particular nutrient. This was achieved by evaporating a solution of the individual nutrients in the presence of a predetermined amount of beads. These beads released nutrients, generating a steep gradient in their vicinity. As with prior experiments (Bondoc, Heuschele, et al., 2016), dSi‐starved cells of the *S. robusta* isolate used in this study were attracted to dSi‐loaded beads within a 10 min observation time (Figure 2). Using the same methods, we tested the attraction of dP and dN‐starved cells to beads loaded with phosphate, nitrate, or ammonium. While dP‐starved cells were attracted to dP‐loaded beads (0.72 nmol dP per bead) within ~20 min (Figure 2, Movie S1), dN‐starved cells did not orient toward dN‐loaded beads (0.54 nmol dN per bead), regardless of the dN form (NO_3_^−^, NH_4_^+^) offered (Figure 2, Movies S2 and S3).

### 3.3 Chemoattraction to dP‐loaded beads

**Figure 3:**
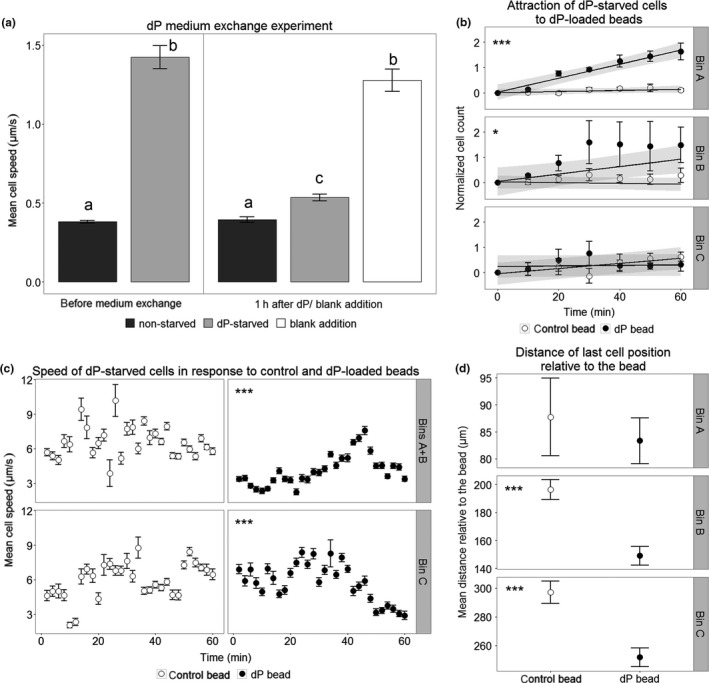
Response of dP‐starved *S. robusta* to bulk dP and dP‐loaded beads. (a) Cells grown in dP‐depleted medium for 5 days have higher mean speed than cells grown in dP‐repleted medium (*p *< 0.001). The cell speed dropped 1 hr after dP addition to starved cells (*p *< 0.001), whereas blank addition of dP‐depleted medium did not affect motility (*p* = 0.160). Data points are presented as mean ± *SEM* of tracked cells from 30‐s movies of each treatment (*n* = 3, *n*_(cells/movie)_ = 100–300). Statistical analysis is linear mixed effects modeling (LME) with pairwise Tukey's honest significance difference (HSD), detailed statistical analysis can be found in Table S3. (b) Normalized cell counts (±*SEM*) showed a significant increase in cell density over time in dP‐loaded beads for all bins compared to the control. A value of zero depicts the population mean. Cells exposed to dP‐loaded beads showed significantly increasing trend over time while the control showed a constant mean value of 0, indicating a stable population mean over time in all bins (linear mixed effects modeling, *n* = 3 movies, for Bin A: *p* < 0.001, Bin B: *p* = 0.0097, and Bin C: *p* = 0. 0189, Table S4). Both Bins A and B showed constant increase in cells over time with Bin B reaching a steady influx of cells after 30 min. This demonstrates cell migration toward the inner bins. The overlaid shaded area shows the LME model fit with 95% confidence intervals. Detailed statistical analysis can be found in Table S4. (c) The mean cell speeds (±*SEM*) over intervals of 2 min were taken for 1 hr from track data of each treatment (*n* = 3, *n*_(cells/movie)_ = 15). Bins A and B were combined to reach sufficient data points. The log + 1 transformed mean speeds were fitted using a generalized additive mixed modeling (GAMM) approach for each bin. Cell speed increases over time in Bins A + B around dP beads, reaching a peak at ~40 min, coinciding with higher cell densities accumulating around the bead (*p*_control bead_ = 0.598, *p*_dP bead_ < 0.001). For Bin C, peak speeds were evident around 25–40 min, wherein cells presumably moved toward the dP gradient source (*p*_control bead_ = 0.193, *p*_dP bead_ < 0.001). Detailed statistical analysis can be found in Table S6 and fitted cubic splines of the model in Figure S2. (d) The mean distance (±*SEM*) was determined from the last coordinate position of the cell against the coordinate position of the bead (*n* = 3, *n*_(cells/movie)_ = 15). Both Bins B and C showed lower mean distance of cells exposed to the dP‐loaded bead compared to controls (pairwise least square means, *n* = 3 movies, for Bin A: *p* = 0.6791, Bin B: *p *< 0.001, Bin C: *p *< 0.001, Table S5). This underscores the inward migration of cells. Detailed statistical analysis can be found in Table S5

To characterize the behavioral response of *S. robusta* to dP, we first determined if cell motility is regulated by dP availability (Figure 3a). After 5 days of dP starvation, starved cells moved three times faster than nonstarved cells. Clearly, this change in motility is resource dependent, as 1 hr after addition of dP to such starved cultures, cell motility significantly decreased to levels comparable to nonstarved cultures. The control treatment with the addition of dP‐deplete medium to starved cells did not result in significantly altered cell speed (Figure 3a, Table S3). This marked change in speed in response to dP is a hallmark of orthokinesis, wherein the speed is regulated by the concentration of the chemical cue that the organism encounters (Amsler & Iken, 2001). To characterize the response to dP gradients, we analyzed the movement of dP‐starved cells and their orientation toward dP‐loaded beads by video monitoring and cell tracking. To allow comparison of the datasets, we selected similar general settings compared to previous investigations on the behavioral response to dSi and attraction pheromones (Bondoc, Heuschele, et al., 2016; Bondoc, Lembke, et al. 2016). Movement of dP‐starved cells exposed to dP‐loaded (Movie S1) and control (Movie S4) beads was recorded over 1 hr. To analyze cell behavior in dP gradients, the observation area (total radius: 336 μm) was divided into three equidistant concentric circles called bins A–C with the bead in the middle (Figure 2). Within ca. 7 min, the dP‐loaded bead generated a gradient with a local concentration of 45 μM at the surface of the bead to 3 μM at the limit of the observation area (Figure S1). Accumulation of *S. robusta* around the bead was evident after ca. 20 min exposure (Figure 2, Table S4). Compared to the control, an increasing cell density over the entire observation time was observed for Bin A (Figure 3b). In Bin B, cell density increased as well during the first 30 min and later a plateau phase was reached (30–60 min). In Bin C, cell density did not statistically differ from the control (Figure 3b). These data indicate an overall migration of cells into the observation area over the entire experiment. Additionally, the mean distance relative to the bead of cells in Bins B and C decreased, confirming that exposure to dP triggers chemoattraction in a tactic mechanism (Figure 3d, Table S5). Cells in proximity to dP‐loaded beads increased their motility, doubling their speed after 45 min exposure. Meanwhile, cell speeds in the control treatment were variable and overall lower with no distinct pattern throughout the observation period (Figure 3c, Figure S2, Table S6). The observed behavior implies that cells adapt toward a dP source by regulating their speed and by directing their movement toward it through simultaneous taxis and kinesis.

## 4 DISCUSSION

### 4.1 Physiology of nutrient depletion

We observed different physiological and behavioral responses of *S. robusta* under nitrogen, phosphorous, or silicate limitation. Starvation and recovery data illustrate the sensitivity of *S. robusta* to dSi starvation. Under limitation of this nutrient no further increase in cell counts is observed, but growth recovers rapidly upon dSi resupplementation (Figure 1). A similar pattern can be observed in centric diatoms, as shown, for example, for *Thalassiosira weissflogii* (De La Rocha & Passow, 2004; De La Rocha, Terbruggen, Volker, & Hohn, 2010). This immediate response to dSi starvation can be explained by the physiological function of the nutrient. Cells during cell division form new silica valves by biomineralization. These minerals represent an irreversible sink for dSi and cannot be remobilized for alternative use (De La Rocha et al., 2010). On the other hand, the effect of dP and dN starvation manifests slower. Here stored reservoirs of the nutrients might be mobilized and utilized during cell division. The more flexible use of these nutrients has also been shown for the planktonic *T. weissflogii* and *Thalassiosira pseudonana* that grow comparatively well even when starved (De La Rocha et al., 2010; Martin, Van Mooy, Heithoff, & Dyhrman, 2011). Other diatoms such as the tychoplanktonic *Cylindrotheca fusiformis* and *Cylindrotheca closterium* and the benthic *Halamphora luciae* exhibit substantially reduced growth rates under dN and dP starvation (Alcoverro, Conte, & Mazzella, 2000; Daglio, Maidana, Matulewicz, & Rodriguez, 2016; Urbani, Magaletti, Sist, & Cicero, 2005). *S. robusta* falls in the first class still exhibiting high growth rates even when dP starved, which might be attributed to efficient utilization of stored dP in the form of polyphosphates or recycled phospholipids (Dyhrman et al., 2012; Martin et al., 2011; Van Mooy et al., 2009). Nitrogen‐limited diatoms can compensate starvation of nitrate by utilizing alternative nitrogen sources such as ammonium, urea, and the free amino acids glutamine and arginine (Jauffrais et al., 2016). They can also utilize intracellular reserves and reuse dN from plastid proteins and polar lipids (Levitan et al., 2015). In the case of *S. robusta*, such compensation is strikingly documented by the continued growth under dN limitation albeit at lower rates. Even after 12 days of starvation, survival was observed (data not shown). This metabolic reorganization can significantly decrease the photosynthetic energy conversion of dN‐starved cells, which is essential for cell division (Geider, Laroche, Greene, & Olaizola, 1993; Levitan et al., 2015). Thus, recovery upon nutrient addition is slow. As dP‐starved diatoms only experience a moderate reduction in photosystem efficiency (Geider et al., 1993), they recover faster than dN‐starved cells. In contrast, dSi is only crucial for cell wall development, and limiting scenarios do not hamper carbon metabolism and nutrient uptake (Dell'Aquila et al., 2017). Thus, dSi‐starved cells reside in a better physiological state than the other two treatments and are ready to resume cell cycle once they encounter dSi. Earlier model simulations complemented with experimental data of planktonic diatoms confirmed a more rapid recovery of dSi‐starved cells compared to dN‐starved cells (De La Rocha & Passow, 2004; De La Rocha et al., 2010; Flynn & Martin‐Jezequel, 2000).

### 4.2 Orientation within nutrient gradients

We have previously shown that dSi‐starved *S. robusta* cells exhibit an active searching behavior to gradients of dSi, a result that is confirmed with the *S. robusta* cell line investigated in this contribution (Bondoc, Heuschele, et al., 2016). A similar but comparably slower response leading to chemoattraction within gradients of dP was observed in phosphorous‐limited cells. In contrast, dN gradients did not trigger any observable searching behavior (Figure 2). This difference might be interpreted in the light of the fact that both, dSi as well as dP, will occur as patchy and slow‐diffusing resources as they are released from mineral sources in the sediment (Karl, 2014). Such gradient‐like hotspot conditions are required for the evolution of an efficient foraging mechanism based upon chemoattraction within a gradient. Nitrogen, however, is rather diffusing into biofilms from the overlying water column and because of its high solubility and diffusivity does not occur in slow‐diffusing gradients (Longphuirt et al., 2009). As such, we propose that active searching behavior is limited to nutrients released from sediment particles as mineral sources that are often within the same size range as the motile cells (Brehm, Gorbushina, & Mottershead, 2005).

As diatoms can be outcompeted by bacteria in taking up dP, the observed directed dP‐foraging mechanism might allow them to compensate for this disadvantage by the location of and accumulation in hotspots (Thingstad, Skjoldal, & Bohne, 1993). Chemotaxis to dP sources has till now only been observed in two planktonic life forms, namely, the dinoflagellagelate *Chattonella antiqua* (Ikegami, Imai, Kato, & Ohtake, 1995) and the bacterium *Thalassospira* sp. (Huetz, Schubert, & Overmann, 2011).

The lack of attraction to dN is surprising as capabilities for dN resource location has been reported for several other aquatic microorganisms like marine and lake water prokaryotes (Dennis, Seymour, Kumbun, & Tyson, 2013; Willey & Waterbury, 1989). Within microeukaryotes, the flagellated planktonic chlorophyte *Dunaliella tertiolecta* is attracted to NH_4_^+^ ions (Sjoblad, Chet, & Mitchell, 1978) and vegetative pregametes of *Chlamydomonas reinhardtii* also exhibit chemotaxis toward NH_4_^+^ (Ermilova, Zalutskaya, Lapina, & Nikitin, 2003; Sjoblad & Frederikse, 1981), NO_3_^−^ (Ermilova, Zalutskaya, & Lapina, 2009), and NO_2_^‐^ (Ermilova & Zalutskaya, 2014). However, as diatoms and green algae respond fundamentally different to dN starvation, the situation might not be comparable. Under nitrogen limitation, green algae increase their carbon stores to produce lipids while diatoms remobilize carbon sources (Alcoverro et al., 2000; Daglio et al., 2016; Guerrini, Cangini, Boni, Trost, & Pistocchi, 2000; Hockin, Mock, Mulholland, Kopriva, & Malin, 2012). As diatom movement is mediated by the excretion of mucus, such metabolic reprogramming might reduce the tendency for displacement and explain the lack of dN responsiveness. Alternatively, even if patchy inorganic sources (NH_4_^+^, NO_3_^−^) would be present, diatoms would rather slowly benefit from their acquisition as indicated by the slow recovery (Figure 1) and thus no efficient utilization of temporal gradients could take place.

### 4.3 Motility patterns of dP attraction

The speed of dP‐starved *S. robusta* is under the influence of the bulk phosphate concentration as well as of diffusing gradients of this resource. The increased motility in dP‐depleted medium might be explained as an increased readiness to relocate under adverse conditions, as it was also observed in the case of dSi depletion (Bondoc, Heuschele, et al., 2016). In a dP gradient, movement during the finding process is adjusted by increasing speed, as well as by taking more or longer forward steps toward the dP‐loaded bead as compared to the control. The observed combined chemokinesis and chemoattraction enable dP‐starved cells to locate dP sources efficiently. Regime changes to universally favorable conditions, simulated by the addition of bulk amounts of dP, lead to an immediate slowing down of the cells while gradients of the resource trigger an increased motility and search behavior. The finding of orientation within a gradient indicates that cells can not only perceive the resource but, moreover, also determine concentration changes during movement.

Although the general patterns of the behavioral response of *S. robusta* to dP, dSi, and the pheromone diproline are similar (Bondoc, Heuschele, et al., 2016; Bondoc, Lembke, et al. 2016), the response to dP was slower, with chemoattraction starting only after ~20 min exposure, as compared to the fast attraction to the latter two within ~5 min (Bondoc, Heuschele, et al., 2016; Bondoc, Lembke, et al. 2016). As the diffusibility of dP and dSi is similar (Hensen, Landenberger, Zabel, & Schulz, 1998), this variable response time could be due to differences in nutrient sensing and uptake mechanisms and how this activates motility in the cells under limitation.

In conclusion, nutrient‐limited *S. robusta* show different response patterns to the addition of the inorganic nutrients dN, dP, and dSi. Principles of chemokinetic and chemotactic movement toward point sources of the inorganic resources dSi, dP, and the organic sex pheromone are similar (Bondoc, Heuschele, et al., 2016; Bondoc, Lembke, et al. 2016). However, the response toward dP is slower, suggesting different perception and signaling pathways. In the benthos, diatoms compete with surrounding organisms for nutrients. While dSi usage is limited to mostly diatoms and might not affect the other microbes in the environment, the observed dP localization has the potential to provide an advantage over competing bacteria that lack such capabilities.

## ACKNOWLEDGMENTS

The authors acknowledge the Volkswagen Foundation, the Max Planck Institute for Chemical Ecology, the IMPRS Exploration of Ecological Interactions with Molecular and Chemical Techniques, the Richard‐Winter‐Stiftung, the International Leibniz Research School for Microbial and Biomolecular Interactions, the CRC1127 ChemBioSys, the Flemish Research foundation project TG.0374.11N, and the Ugent research grants 01/04611 and BOF15/GOA/17 for financial support.

## CONFLICT OF INTEREST

None declared.

## DATA ACCESSIBILITY

The datasets generated and analyzed during this study are available from the corresponding author K.G.V.B on reasonable request.
