# Supplementary material for: Selective chemoattraction of the benthic diatom Seminavis robusta to phosphate but not to inorganic nitrogen sources contributes to biofilm structuring
Source: Microbiologyopen. 2018 Jul 22;8(4):e00694. doi: 10.1002/mbo3.694 (PMC6460271; doi:10.1002/mbo3.694)
Supplement: Supplementary file 1 [file MBO3-8-e00694-s001.docx]

**Selective chemoattraction of the benthic diatom *Seminavis robusta* to phosphate and silicate but not nitrate contributes to biofilm structuring**

Karen Grace V. Bondoc^1, 2, 4, *^, Christine Lembke^1, 4^, Wim Vyverman^3^, Georg Pohnert^1, 2, *^

^1^Institute for Inorganic and Analytical Chemistry, Bioorganic Analytics, Friedrich-Schiller-Universität Jena, Lessingstrasse 8, D-07743 Jena, Germany

^2^Max Planck Institute for Chemical Ecology, Hans-Knöll-Str. 8, D-07745 Jena

^3^Laboratory of Protistology and Aquatic Ecology, Department of Biology, University Gent, Krijgslaan 281 S8, 9000 Gent, Belgium

^4^ Co-first author

* Correspondence: [karen.bondoc@gmail.com](mailto:karen.bondoc@gmail.com) (K.G.V.B.); [Georg.Pohnert@uni-jena.de](mailto:Georg.Pohnert@uni-jena.de) (G.P.)

**Supporting Information**

**SI Materials and Methods**

***Diffusion of dP from the bead***

We determined the diffusion gradient formed by the dP-loaded beads for 1 h through a combination of experimental and computational approach, as we have previously done for dSi and diproline gradient determination (S1, S2). The total flux of dP diffused out (*i*) was calculated by exposing 100 mg dP-loaded beads or control beads prepared as described previously with 50 ml ASW. After incubation at room temperature for 1 h the medium was filtered (Filtropur 0.2, Sarstedt, Nümbrecht, Germany) and stored at –20 °C until measurement. The phosphate concentrations were determined as described before through standard colorimetric methods. We calculated *i* using the formula from Barbara and Mitchell (S3).

*C_(r,t)_ = i/4πrD*

where *i* is the total diffusive flux of dP, *r* is the radius of the bead (55 µm), *t* is the time until steady state was reached, and *D* is the diffusivity constant for small molecules (10^-5^ cm^-2^ s^-1^) (S4). We then determined the steady state gradient *C* by using the computed *i* (8.16^-15^ ± 6.42^-16^ mol s^-1^) and modifying √r as the radius of the observation area (336 µm) to correct for the shape of the gradient in a flat chamber (S5). The time to steady state was determined as the time *≥d^2^/D* where d is the diameter of the whole observation area (672 µm). Supplementary Figure 2 depicts the formed gradient.

**SI Figures**

**
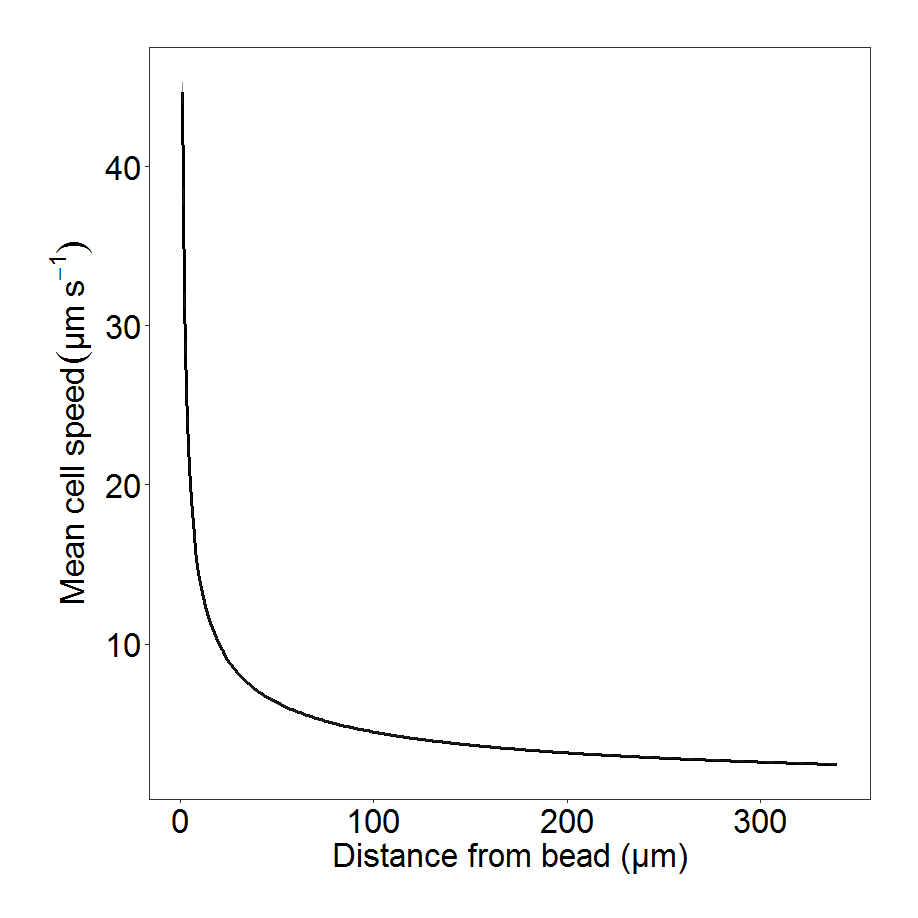
**

**Figure S1.** dP gradients were formed until the edge of the observation area in ~450 s. Bin A showed steep concentrations of dP ranging from ~44 µM from the surface of the bead to ~4 µM, whereas, Bins B and C have a constant 2-4 µM.


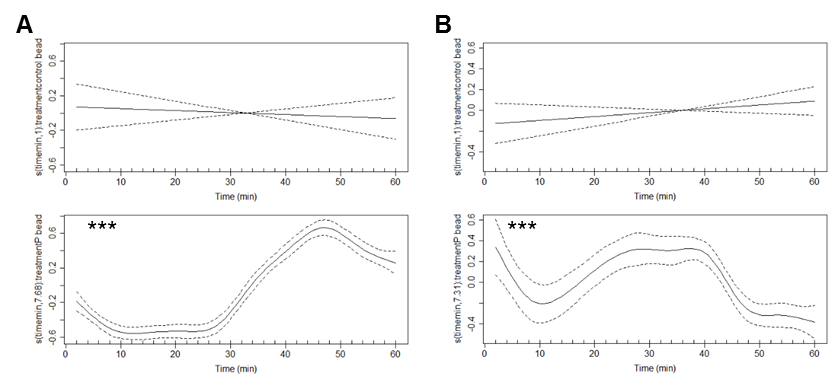


**Figure S2.** Fitted cubic splines of cell speed for the dP attraction experiment (upper panel: control, lower panel: dP-loaded bead) for Bins A+B (a) and Bin C (b) over 60 min. The y-axis shows the optimal smoothing knots (i.e. the number of connections for each data point over time). GAMM centers the mean of knots to 0. For the controls of both Bins A+B and Bin C, a straight line could be observed, suggesting that there were no observed changes on the mean speed over time. On the other hand, dP-loaded beads induced cells increased speed around ~30 min and ~15 min for both Bins A+B and Bin C, respectively, implying an orthokinetic response.

**SI Tables**

**Table S1.** Detailed statistical parameters for each model fit of the corresponding figures mentioned.

| Figure | Experimental Set | Supplementary Result Table | Sample Size (n) | Model | Random Factor | Correlation Structure | Variance Structure | Extra processing |
| --- | --- | --- | --- | --- | --- | --- | --- | --- |
| 1b and 1c | Nutrient starvation and recovery | Table 2 | n_replicates_ = 3 | LME with post-hoc test (Tukey’s HSD):  lncells^a^ ~ treatment*day | Replicate ID | Replicate ID and treatment | - | Outliers were removed |
| 2a | dP medium exchange experiment | Table 3 | n_movies_ = 3,  n_cells/movie_ = 100-300 | LME with post-hoc test (Tukey’s HSD):  Vlog^b^ ~ treatment | Track ID | - | Treatment | 1^st^ 30 s of video was removed, only complete 30 s data were used for analysis |
| 2b | dP-attraction: cell counts | Table 4 | n_movies_ = 3 | LME (per bin):  cellsBase^c^ ~ treatment*time | Replicate ID | Replicate ID and treatment | Replicate ID | - |
| 2c | dP-attraction: speed | Table 5 | n_movies_ = 3,  n_cells/movie_ = 15 | GAMM (per bin):  Vlog ~ smoothed factor interaction of time*treatment penalized with cubic regression splines | Track ID | Track ID and treatment | Treatment | Bins A+B were combined, Outliers were removed |
| 2d | dP-attraction: sum distance | Table 6 |  | LME (all data) with post-hoc test (LS-means):  distance^d^ ~ treatment*bin | Track ID | Track ID and treatment | Bin | - |

^a^lncells = log normal transformation of cell counts

^b^Vlog = log+1 transformation of speed

^c^cellsBase = Z standardized cell count calculated per treatment using the formula: *Z= (X-µ)/σ*, where µ is mean, X is score and σ is standard deviation

^d^distance = sum Euclidean distance from all track IDs calculated from the first and last coordinate position of the cell

**Table S2.** Pair-wise comparisons using Tukey’s HSD of log normal cell counts of batch cultures during nutrient starvation and recovery. Cultures in full-nutrient ASW acted as control.

| Pair-wise comparisons | Estimate | Std. error | z-value | Pr(>\|z\|) |
| --- | --- | --- | --- | --- |
| control – dSi-starvation | 1.94234 | 0.13590 | 14.293 | < 0.001 *** |
| control – dN-starvation | 0.41632 | 0.13590 | 3.063 | 0.03577 * |
| control – dP-starvation | -0.28641 | 0.14021 | -2.043 | 0.38737 |
| control – dSi-recovery | 1.22408 | 0.13895 | 8.809 | < 0.001 *** |
| control – dN-recovery | 0.31864 | 0.13895 | 2.293 | 0.24711 |
| control – dP-recovery | -0.22694 | 0.13895 | -1.633 | 0.66049 |
| dSi-starvation – dN-starvation | -1.52602 | 0.13590 | -11.229 | < 0.001 *** |
| dSi-starvation – dP-starvation | -2.22875 | 0.14021 | -15.896 | < 0.001 *** |
| dSi-starvation – dSi-recovery | 0.71826 | 0.13895 | 5.169 | < 0.001 *** |
| dSi-starvation – dN-recovery | -1.62370 | 0.13895 | -11.685 | < 0.001 *** |
| dSi-starvation – dP-recovery | -2.16928 | 0.13895 | -15.611 | < 0.001 *** |
| dSi-recovery – dN-recovery | -0.90544 | 0.14194 | -6.379 | < 0.001 *** |
| dSi-recovery – dP-recovery | -1.45102 | 0.14194 | -10.222 | < 0.001 *** |
| dSi-recovery – dN-starvation | -0.80776 | 0.13895 | -5.813 | < 0.001 *** |
| dSi-recovery – dP-starvation | -1.51049 | 0.14318 | -10.550 | < 0.001 *** |
| dN-starvation – dP-starvation | 0.70274 | 0.14021 | 5.012 | < 0.001 *** |
| dN-starvation – dN-recovery | 0.09768 | 0.13895 | 0.703 | 0.99246 |
| dN-starvation – dP-recovery | 0.64326 | 0.13895 | 4.629 | < 0.001 *** |
| dN-recovery – dP-starvation | 0.60505 | 0.14318 | 4.226 | < 0.001 *** |
| dN-recovery – dP-recovery | 0.54558 | 0.14194 | 3.844 | 0.00243 ** |
| dP-starvation – dP-recovery | -0.05948 | 0.14318 | -0.415 | 0.99961 |

Signif. codes: 0 ‘***’ 0.001 ‘**’ 0.01 ‘*’ 0.05 ‘.’ 0.1 ‘ ’ 1 (Adjusted p values reported -- single-step method)

**Table S3.** Pair-wise comparisons using Tukey’s HSD of speed (log+1 transformed) of dP-starved and non-starved cultures before and 1 h after addition of dP or blank.

| Pair-wise comparisons | Estimate | Std. error | z-value | Pr(>\|z\|) |
| --- | --- | --- | --- | --- |
| before medium exchange | | | | |
| starved – non-starved | 0.426028 | 0.025522 | 16.693 | <1e-04 *** |
| after medium exchange |  |  |  |  |
| starved – starved+dP | 0.367292 | 0.026629 | 13.793 | <1e-04 *** |
| starved – starved+blank | 0.074003 | 0.033724 | 2.194 | 0.160 |
| starved – non-starved+dP | 0.435928 | 0.026118 | 16.691 | <1e-04 *** |
| starved+dP – starved+blank | 0.293289 | 0.024589 | 11.928 | <1e-04 *** |
| starved+dP – non-starved+dP | 0.068636 | 0.012225 | 5.614 | <1e-04 *** |
| starved+dP – non-starved | 0.058736 | 0.010893 | 5.392 | <1e-04 *** |
| starved+blank - non-starved+dP | 0.361925 | 0.024035 | 15.058 | <1e-04 *** |
| non-starved – starved+blank | 0.352025 | 0.023385 | 15.053 | <1e-04 *** |
| non-starved – non-starved+blank | 0.009900 | 0.009576 | 1.034 | 0.821 |

Signif. codes: 0 ‘***’ 0.001 ‘**’ 0.01 ‘*’ 0.05 ‘.’ 0.1 ‘ ’ 1 (Adjusted p values reported -- single-step method)

**Table S4.** Linear mixed effects (LME) model results on count data (Z-standard score transformed) on the dP attraction experiment (control bead vs. dP-loaded bead) for individual bins.

|  | numDF | denDF | F-value | p-value |
| --- | --- | --- | --- | --- |
| Bin A | | | | |
| (intercept) | 1 | 34 | 22.06955 | <.0001 |
| treatment | 1 | 4 | 42.56985 | 0.0028 |
| time | 1 | 34 | 17.67461 | 0.0002 |
| treatment:time | 1 | 34 | 36.13671 | <.0001 |
| Bin B | | | | |
| (intercept) | 1 | 34 | 0.767932 | 0.3870 |
| treatment | 1 | 4 | 3.81304 | 0.1226 |
| time | 1 | 34 | 0.0124 | 0.9120 |
| treatment:time | 1 | 34 | 7.511128 | 0.0097 |
| Bin C | | | | |
| (intercept) | 1 | 34 | 3.323564 | 0.0771 |
| treatment | 1 | 4 | 0.001258 | 0.9734 |
| time | 1 | 34 | 7.093012 | 0.0117 |
| treatment:time | 1 | 34 | 6.072297 | 0.0189 |

**Table S5.** Pair-wise comparisons using least square (LS) means of sum distance of cells on the dP attraction experiment (control bead vs. dP-loaded bead) for individual bins.

|  | Estimate | Std. error. | DF | t.ratio | p-value |
| --- | --- | --- | --- | --- | --- |
| Bin A | 3.050294 | 7.346313 | 80 | 0.415 | 0.6791 |
| Bin B | 45.93821 | 9.744273 | 80 | 4.714 | <.0001 *** |
| Bin C | 43.24334 | 9.911365 | 80 | 4.363 | <.0001 *** |

Signif. codes: 0 ‘***’ 0.001 ‘**’ 0.01 ‘*’ 0.05 ‘.’ 0.1 ‘ ’ 1 (Adjusted p values reported -- single-step method)

**Table S6.** General additive mixed model (GAMM) results on speed data (log+1 transformed) on the dP-attraction experiment (control bead vs. dP-loaded bead) for individual bins. Bins A and B were combined because of insufficient data points in Bin A.

|  | edf | Ref.df | F | p-value |
| --- | --- | --- | --- | --- |
| Bins A+B | | | | |
| s(time):controlbead | 1.000 | 1.000 | 0.278 | 0.598 |
| s(time):dPbead | 7.684 | 7.684 | 60.811 | <2e-16 |
| Bin C | | | | |
| s(time):controlbead | 1.000 | 1.000 | 1.697 | 0.193 |
| s(time):dPbead | 7.311 | 7.311 | 14.502 | <2e-16 |

**SI Movies**

**Movie S1.** This 1 h movie shows the chemoattraction of dP-starved cells to a dP-loaded bead. Cells began to move towards the bead within ~20 min of bead exposure. The video speed was accelerated 50 times and the scale bar indicates 100 µm. Time stamp denotes min:s.

**Movie S2.** This 1 h movie shows a dN-loaded bead (NH_4_^+^) eliciting no response from dN-starved cells. The video speed was accelerated 50 times and the scale bar indicates 100 µm. Time stamp denotes min:s.

**Movie S3.** This 1 h movie shows a dN-loaded bead (NO_3_^-^) eliciting no response from dN-starved cells. The video speed was accelerated 50 times and the scale bar indicates 100 µm. Time stamp denotes min:s.

**Movie S4.** This 1 h movie shows the control bead inducing no response from dP-starved cells. The video speed was accelerated 50 times and the scale bar indicates 100 µm. Time stamp denotes min:s.

**Author contributions**

Conceptualization, K.G.V.B., C.L., and G.P.; Methodology, K.G.V.B. and C.L.; Formal Analysis, K.G.V.B; Investigation, K.G.V.B. and C.L.; Resources, G.P. and W.V.; Data Curation, K.G.V.B. and G.P.; Writing - Original Draft, K.G.V.B., C.L., and G.P., Writing - Review & Editing, K.G.V.B., C.L., W.V., and G.P.: Visualization, K.G.V.B. and C.L.; Supervision, G.P.

**SI References**

S1 Bondoc KGV, Heuschele J, Gillard J, Vyverman W, & Pohnert G (2016) Selective silicate-directed motility in diatoms. *Nat. Com.* 7:10540.

S2 Bondoc KGV, Lembke C, Vyverman W, & Pohnert G (2016) Searching for a mate: pheromone-directed movement of the benthic diatom *Seminavis robusta*. *Microb. Ecol.* 72(2):287-294.

S3 Barbara GM & Mitchell JG (2003) Marine bacterial organisation around point-like sources of amino acids. *FEMS Microbiol. Ecol.* 43(1):99-109.

S4 Berg HC (1993) *Random walks in biology* (Princeton University Press).

S5 Blackburn N, Fenchel T, & Mitchell J (1998) Microscale nutrient patches in planktonic habitats shown by chemotactic bacteria. *Science* 282(5397):2254-2256.
